# mTOR Signalling Pathway: A Potential Therapeutic Target for Ocular Neurodegenerative Diseases

**DOI:** 10.3390/antiox11071304

**Published:** 2022-06-29

**Authors:** Yipin Wang, Nicholas Siu Kay Fung, Wai-Ching Lam, Amy Cheuk Yin Lo

**Affiliations:** Department of Ophthalmology, School of Clinical Medicine, Li Ka Shing Faculty of Medicine, The University of Hong Kong, Hong Kong 999077, China; u3005138@connect.hku.hk (Y.W.); nfung@hku.hk (N.S.K.F.)

**Keywords:** mTOR, AMD, DR, glaucoma, oxidative stress, hypoxia, inflammation, ROS, rapamycin, clinical trial

## Abstract

Recent advances in the research of the mammalian target of the rapamycin (mTOR) signalling pathway demonstrated that mTOR is a robust therapeutic target for ocular degenerative diseases, including age-related macular degeneration (AMD), diabetic retinopathy (DR), and glaucoma. Although the exact mechanisms of individual ocular degenerative diseases are unclear, they share several common pathological processes, increased and prolonged oxidative stress in particular, which leads to functional and morphological impairment in photoreceptors, retinal ganglion cells (RGCs), or retinal pigment epithelium (RPE). mTOR not only modulates oxidative stress but is also affected by reactive oxygen species (ROS) activation. It is essential to understand the complicated relationship between the mTOR pathway and oxidative stress before its application in the treatment of retinal degeneration. Indeed, the substantial role of mTOR-mediated autophagy in the pathogenies of ocular degenerative diseases should be noted. In reviewing the latest studies, this article summarised the application of rapamycin, an mTOR signalling pathway inhibitor, in different retinal disease models, providing insight into the mechanism of rapamycin in the treatment of retinal neurodegeneration under oxidative stress. Besides basic research, this review also summarised and updated the results of the latest clinical trials of rapamycin in ocular neurodegenerative diseases. In combining the current basic and clinical research results, we provided a more complete picture of mTOR as a potential therapeutic target for ocular neurodegenerative diseases.

## 1. Introduction

The mammalian target of rapamycin (mTOR), also known as the mechanistic target of rapamycin, is a 289-kDa serine/threonine kinase that belongs to the phosphoinositide 3-kinase (PI3K)-related kinase family and is highly conserved in evolution. It plays a central role in cell growth, cell survival autophagy, and metabolism via two distinct protein complexes of mTOR complex 1 (mTORC1) and mTOR complex 2 (mTORC2) [1]. mTOR also integrates with other signalling pathways, including PI3K/AKT, tuberous sclerosis complex subunit 1 (TSC1)/tuberous sclerosis complex subunit 2 (TSC2)/Rheb, LKBL/adenosine 5′-monophosphate-activated protein kinase (AMPK), and VAM6/Rag GTPases, by affecting transcription and protein synthesis [2]. Dysregulation of the mTOR signalling pathway is involved in many diseases, such as cancers, neurodegenerative diseases, and diabetes mellitus [3,4,5,6]. Clinical data show that the mTOR signal is abnormally overactivated in nearly 30% of cancers and is one of the most frequently altered cascades in human cancers [7].

Recently, more and more studies are focused on the potential therapeutic effects of mTOR inhibitors in neurodegenerative diseases that are linked with oxidative stress. In a rat model of Alzheimer’s disease (AD) induced by zinc injection, inhibition of mTOR by rapamycin attenuated zinc-induced tau phosphorylation and elevated levels of oxidative stress, as well as the synaptic impairment and decrease in cognitive function [8]. In a Parkinson’s disease (PD) model induced by 6-hydroxydopamine (6-OHDA), pathogenic oxidative stress increased the negative mTOR regulator tuberous sclerosis complex 2 (TSC2) and increased autophagy in dopaminergic neurons, implicating that mTOR is a potential intervention target for oxidative-stress-induced dysfunctional autophagy in PD [9]. The retina is a part of the central nervous system; it contains complex neural circuitry and transduces the converted electrical potentials to the brain [10]. The neuroprotective roles of rapamycin may be a novel therapeutic pathway in ocular neurodegenerative diseases, such as diabetic retinopathy (DR), age-related macular degeneration (AMD), and glaucoma, which share common pathophysiological mechanisms, especially increased and prolonged oxidative stress, which would ultimately result in retinal neuronal death [11,12,13,14]. Recently, a large number of studies have been conducted to elucidate the neuroprotective role of rapamycin and its underlying mechanism(s) in the treatment of ocular degenerative diseases [15,16,17,18,19,20,21,22,23,24,25,26,27]. For instance, a study has shown that rapamycin ameliorated high-glucose-induced ROS formation and inflammatory injury in retinal pigment epithelial (RPE) cells [28]. 

In this review, we introduced the mTOR signalling pathway and its role in ocular neurodegenerative diseases under oxidative stress, trying to highlight and summarise the current understanding of the mechanisms of mTOR inhibitors, especially rapamycin and its analogues, in different retinal models, including DR, AMD, and glaucoma. In updating the latest basic research findings and clinical trial results, we attempted to shed light on the novel therapeutic strategies of mTOR in ocular degenerative diseases. 

## 2. mTOR Signalling Pathway

mTOR is part of the catalytic subunit in two structurally and functionally distinct complexes, known as mTOR complex 1 (mTORC1) and mTOR complex 2 (mTORC2) [1]. mTORC1 consists of five components: mTOR; regulatory-associated protein of mTOR (Raptor); mammalian lethal with Sec13 protein 8 (mLST8, also referred as GβL); proline-rich AKT substrate 40 kDa (PRAS40); and DEP-domain-containing mTOR-interacting protein (Deptor) [29]. Raptor has been reported to function as a scaffolding protein that controls mTORC1 activity by regulating assembly of the complex and by recruiting substrates for mTOR [30]. The role of mLST8 in mTORC1 function is still unclear; however, it is established that mLST8 is indispensable for mTORC2 integrity and kinase activity [31]. PRAS40 and Deptor have been identified as distinct negative regulators of mTORC1. When the activity of mTORC1 decreases, PRAS40 and Deptor are recruited to the complex and promote the inhibition of mTORC1. It has been reported that PRAS40 regulates mTORC1 kinase activity by direct inhibition of substrate binding [32]. On the other hand, mTORC1 once activated would directly phosphorylate PRAS40 and Deptor, which reduce their physical interaction with mTORC1 and further activate mTORC1. 

Similar to mTORC1, mTORC2 also comprises six different proteins, among which three are identical to those in mTORC1, including mTOR, mLST8, and Deptor [29]. The remaining three components include rapamycin-insensitive companion of mTOR (Rictor), mammalian stress-activated protein kinase interacting protein (mSIN1), and protein observed with Rictor-1 (Protor-1). It is proposed that Rictor and mSIN1 stabilise each other and establish the structural foundation of mTORC2. Rictor also interacts with Protor-1, but the physiological function of this interaction is not yet clear [33]. 

**Figure 1:**
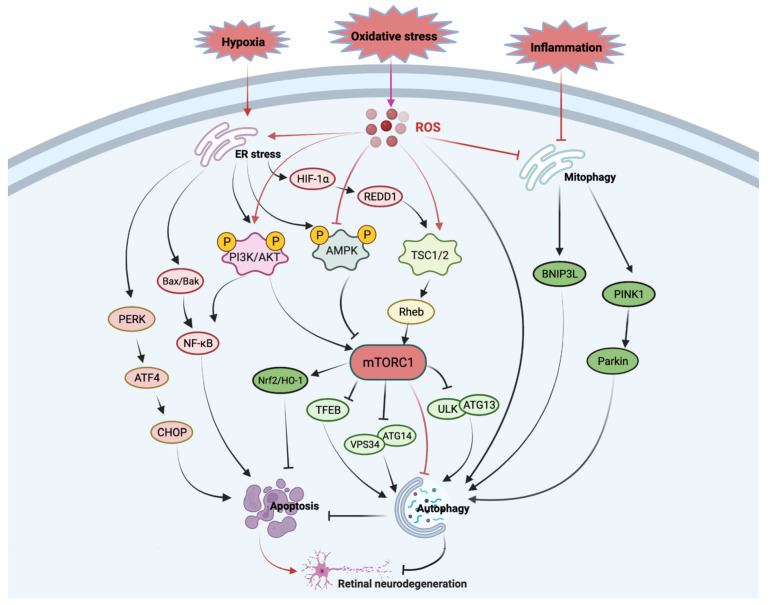
The common mTOR signalling pathway in ocular neurodegenerative diseases. Abbreviations: AMP’, 5’ adenosine monophosphate-activated protein kinase; ATF4, activating transcription factor 4; ATG, autophagy-regulating protease; BNIP3L, adenovirus E1B 19 kDa protein-interacting protein 3-like; CHOP, C/EBP homologous protein; ER, endoplasmic reticulum; HIF-1α, hypoxia-inducible factor 1-alpha; HO-1, heme oxygenase-1; mTORC1, mammalian target of rapamycin complex 1; Nrf2, nuclear factor-erythroid factor 2-related factor 2; PERK, protein kinase R (PKR)-like endoplasmic reticulum kinase; PI3K, phosphoinositide 3-kinase; PINK1, PTEN-induced kinase 1; REDD1, regulated in development and DNA damage responses 1; Rheb, ras homolog enriched in brain; ROS, reactive oxygen species; TFEB, transcription factor EB; TSC, tuberous sclerosis complex; ULK1, unc-51-like kinase 1; VPS34, vacuolar protein sorting 34.

The mTOR signalling pathway integrates both intracellular and extracellular signals and transduces divergent signalling cascades. Apart from intracellular signals, such as cellular energy status and hypoxia stress, extracellular signals that include growth factors, amino acids, and hormones play essential roles in regulating the activity of mTORC1 [34]. Insulin, a pivotal hormone serving to maintain energy balance and glucose homeostasis, initiates a signalling cascade through the insulin receptor (IR), insulin receptor substrate (IRS), class I phosphoinositide 3-kinases (PI3Ks), phosphoinositide-dependent protein kinase 1 (PDK1), and AKT (also known as protein kinase B) [35]. AKT activates mTORC1 through phosphorylation of TSC1/TSC2 and PRAS40 (Figure 1). It has been reported that there is a feedback loop between AKT and mTOCR2 induced by insulin [36].

In addition to AKT, mitogen-activated protein kinase (MAPK) and proinflammatory cytokine TNFα also promote the activation of mTORC1 through inhibiting TSC1/TSC2 [37]. Wnt/β-catenin signalling plays a role in regulating mTORC1 by suppressing glycogen synthase kinase 3β (GSK3-β), which phosphorylates and increases TSC1/2 activity [38]. AMPK is one of the signalling pathways that inhibits the activation of mTORC1 by direct phosphorylation of TSC2, promoting TSC1/2 activity [39]. AMPK can be activated by DNA damage via p53-dependent transcription of Sestrin1/2 [40]. 

Oxidative stress reflects an imbalance in the production of reactive oxygen species (ROS) and the antioxidative capacity in the cell. The retina is susceptible to ROS due to its high energy consumption and exposure to light. ROS is not only a by-product generated in the retinal cells but also a signal transducer involved in the PI3K/AKT/mTOR signalling pathway (Figure 1). The increased level of intracellular ROS activates the kinases, including PKC, MARK, and PI3K, which leads to the amplification of their downstream signalling, respectively [41,42]. ROS can also inhibit the activation of phosphatase and tensin homolog (PTEN), which negatively regulates the synthesis of PIP3, a signalling molecule in the plasma membrane, which plays a role in the activation of AKT [43]. 

In regard to the downstream signalling of mTOR, there are many substrates for mTORC1, such as p70 ribosomal protein S6 kinase (p70S6K) and eukaryotic initiation factor 4E (eIF4E)-binding protein (4EBP), that are responsible for translation control [44]. Other mTORC1 substrates, such as Unc-51-like autophagy activating kinase 1 (ULK1) and ATG13, play essential roles in the regulation of autophagy (Figure 1). Studies have shown that AKT, which regulates mTORC1 activity, is also an mTORC2 substrate. Protein kinase C (PKC), which regulates diverse cellular functions, is an mTORC2 substrate as well [30,45]. 

The mTOR signalling pathway plays a central role in the regulation of autophagy, whereas autophagy maintains retinal homeostasis by removing dysfunctional organelles and unfolded proteins [46]. Hence, mTOR modulates oxidative stress by both direct and indirect mechanisms. A study has shown that autophagy played a neuroprotective role in DR [47]. The autophagic genes beclin-1 and LC3 were moderately up-regulated, which was accompanied with increased phosphorylated AMPK and decreased phosphorylated mTOR in the diabetic retinas [48]. After the inhibition of autophagy by 3-MA in STZ-induced diabetic rats, RGC apoptosis increased when compared with the vehicle-treated group [48]. Studies have also demonstrated that autophagy-deficient cells lacking BECN1, ATG5, or ATG7 caused the accumulation of impaired organelles [49,50]. There is a feedback loop in which autophagy modulates oxidative stress through activating transcription factors, such as NRF2 and p53 [40,51]. 

A recent study quantified the expression of mTORC1- and mTORC2-specific partner proteins in normal adult rat retina, brain, and liver, and further localised these components in normal adult human and mouse retina [52]. They found a relatively higher content of mTORC1, mTORC2, and their components included higher Raptor (mTORC1) and Rictor (mTORC2) in the retina than the brain and liver. The two mTOR complexes may serve distinct purposes within the retina, while the mTORC1 complex is predominantly expressed in retinal ganglion cells (RGCs) and their axons with lower expression in the inner plexiform layer (IPL) and inner nuclear layer (INL); mTORC2 complex proteins, such as Rictor, were mainly found in astrocytes and Müller cells. However, no detectable immunoreactivity of mTORC1- and mTORC2-specific components was found in photoreceptor cells, RPE, and vascular endothelial cells. The composition and topology of mTOR components closely parallel the physiological characteristics of the retina as a tissue of high energy and oxygen consumption and the role of the mTOR signalling pathway in retinal metabolism and homeostasis in response to glucose.

## 3. mTOR Inhibitors

mTOR inhibitors are a class of drugs that inhibit the activity of the serine/threonine-specific protein kinase coded by the *MTOR* gene. This protein kinase belongs to the family of phosphatidylinositol-3 kinase (PI3K)-related kinases (PIKKs) and serves as the catalytic subunit of two multi-protein complexes (mTORC1 and mTORC2) [53] (Table 1). 

The best-established mTOR inhibitors are rapamycin and its analogues (rapalogs). Rapamycin is a good example of the first-generation mTOR inhibitors, which were initially introduced as immunosuppressive drugs and approved by the FDA in 1997 in use for transplant surgery to prevent allograft rejection [63]. Other therapeutic effects, such as anti-cancer activity, angiogenesis, and neuroprotection, were discovered and exploited in the following years [64,65,66]. Rapamycin is a macrolide compound containing two binding moieties for mTOR and FKBP12, respectively. This engagement of the binding moieties has limited the modification of the ATP binding pocket. Thus, further drug development for rapamycin mainly focused on improving its pharmacokinetics and stability due to its low aqueous solubility [67]. Rapalogs were derived and evolved from rapamycin; they have a more favourable pharmacokinetic profile when compared to their parent drug.

**Figure 2:**
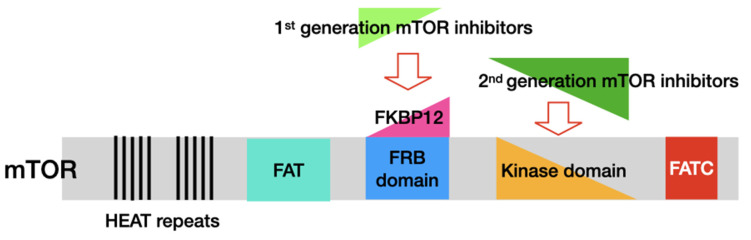
Domains of the mTOR protein and two generations of mTOR inhibitors.

However, they still have the same binding sites for mTOR and FKBP12 as in rapamycin. Their mechanism of action is also identical; they bind with FKBP12 to form a complex, which then binds to the FRB domain of mTOR. Through modulating mTOR formation, both rapamycin and rapalogs inhibit the kinase activity of mTORC1 (Figure 2). Rapalogs include temsirolimus (CCI-779), everolimus (RAD001), ridaforolimus (AP23573), umirolimus, and zotarolimus (ABT-578) [68,69,70,71]. 

mTOR protein is composed of several structural domains, including HEAT repeats and FAT, FRB, and FATC domains. The first-generation mTOR inhibitors bind to FKBP12 and then interact with the FRB domain of mTOR to inhibit mTOR activity. The second-generation mTOR inhibitors are ATP-competitive mTOR inhibitors that act as ATP analogues and bind to the kinase domain of mTOR. Abbreviations: FAT domain, FKBP12-rapamycin-associated protein, ataxia-telangiectasia and transactivation/transformation domain; FATC domain, FAT carboxyterminal domain; FRB domain, FKBP12-rapamycin-binding domain; HEAT, Huntingtin, elongation factor 3 (EF3), protein phosphatase 2A (PP2A), and the yeast kinase TOR1.

**Figure 3:**
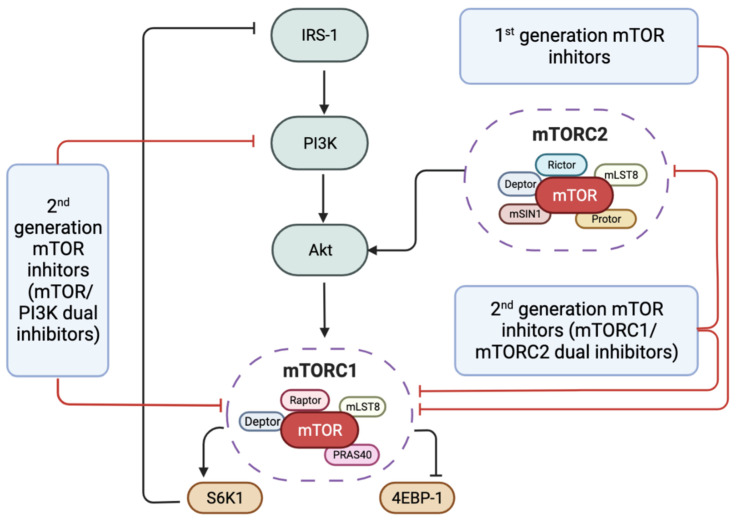
The signalling pathway that is modulated by different generation mTOR inhibitors. Inhibition of mTORC1 results in the suppression of 4E-BP1 and S6K1 phosphorylation. Inhibited S6K1 reduces protein synthesis through phosphorylation of the 40S ribosomal subunit, which has been suggested to decrease the translational efficiency of a class of mRNA transcripts with a 5′-terminal oligopolypirymidine. There is a negative feedback loop in which mTORC1 activation can inhibit the PI3K pathway by S6K1-mediated phosphorylation and degradation of IRS-1, and it fills an important gap in our understanding the underlying mechanisms by which mTORC1 inhibits PI3K-Akt signalling. Abbreviations: IRS-1, insulin receptor substrate 1; PI3K, phosphatidylinositol-3-kinase; AKT, protein kinase B; PRAS40, proline-rich AKT substrate of 40 kDa; mTOR, mammalian target of rapamycin; S6K1, S6 kinase 1; 4E-BP1.

The second-generation mTOR inhibitors are ATP-competitive mTOR kinase inhibitors, which have been developed as two types. They include mTORC1/mTORC2 dual inhibitors, such as Torin1, PP242, and AZD8055, and dual mTOR/PI3K inhibitors, such as PI-103, OSI-027, GSK2126458, and NVP-BEZ235 [72,73,74]. They are developed to compete with ATP in the catalytic domain of mTOR (Figure 2). Compared with rapalogs, which only inhibit mTORC1 activity, the second-generation mTOR inhibitors are designed to target both mTORC1 and mTORC2 and inhibit all the catalytic isoforms of PI3K (Figure 3). As a result of the blockade in the feedback activation of PI3K/AKT signalling in mTORC1, they impose more potent inhibition on the mTOR pathway and stronger induction of autophagy than rapalogs (Table 1) [75]. This new generation mTOR inhibitors has been introduced into clinical trials mainly for the treatment of various cancers [71,76]. 

## 4. mTOR in Ocular Neurodegenerative Diseases

### 4.1. mTOR and Diabetic Retinopathy (DR)

The pathogenesis of DR is complex and multifactorial. Many biochemical mechanisms are involved in the development and progression of DR. For instance, polyol pathway activation, increased advanced glycation end-products (AGEs) formation, activation of PKC, and induction of the hexosamine pathway are related to the pathogenesis of DR [77]. These pathways induce inflammation, oxidative stress, and vascular dysfunction in the retina. Chronic hyperglycaemia in diabetes mellitus favours the production and accumulation of ROS, which leads to oxidative-stress-induced impairment in different cells of the retina, especially the retinal neurons [78]. The classical view is that microvascular alteration is the primary event in the pathogenesis of DR. However, there is growing evidence showing that neurodegeneration occurs in the early stage of DR, which could also be related to the development of microvascular abnormalities [79,80,81,82]. Diabetic retinal neurodegeneration is characterised by reactive gliosis and retinal neuron apoptosis. While RGC and amacrine cell death induced by diabetes-induced apoptosis occur in the early phase of DR, other retinal neurons, such as photoreceptors, also have an increased apoptotic rate [83,84]. Reactive gliosis (glial activation) may also be involved in retinal neurons apoptosis and may be associated with the neurodegenerative process with microvascular disease [85]. In addition to macroglial cells, activated microglia, the main resident immune cells of the retina, and infiltrating monocytes also mediate diabetes-induced inflammation [86]. 

Recently, more and more studies have emphasised the key roles of inflammation, oxidative stress, and autophagy in the pathogenesis of DR. As described above, the mTOR pathway plays a substantial role in regulating autophagy. The activated mTORC1 inhibits autophagy through various steps, including the inhibitory phosphorylation of ULK1 and transcription factor EB (TFEB), which initiates autophagy and promotes lysosomal biogenesis required to degrade the contents of autophagosomes, respectively. LC3B and Beclin-1 are cellular autophagy markers involved in the initial stage of autophagosome formation. Park et al. observed a slight increase in Beclin-1 and the ratio of LC3B-II-to-LC3B-I after 1, 4, and 8 weeks of hyperglycaemia in STZ-induced diabetic rats compared with the non-diabetic rat [48]. Of note, the level of Beclin-1 decreased dramatically, even lower than the basal level, after prolonged hyperglycaemia over 8 weeks. Significant upregulation of phosphorylated AMPK but downregulation of phosphorylated mTOR was also observed in the early stage of DR before 8 weeks of hyperglycaemia. After the inhibition of autophagy using 3-methyladenine (3-MA), apoptosis of RGCs was significantly increased in the diabetic retinas. These results indicated that autophagy induced by hyperglycaemia may act as a survival attempt to rescue RGC apoptosis; however, the insufficient activation of autophagy failed to maintain retinal homeostasis. It suggested that AMPK-activation-induced autophagy may play a neuroprotective role in DR. 

A similar result was also reported in a recent study. Fang et al. found that LC3-II and p62 levels, as well as the phosphorylated proteins in the PI3K/Akt/mTOR signalling pathway in STZ-induced diabetic rats, were increased when compared with the normal control group [87]. This suggested that retinal autophagy was initiated in DR but was inadequate to protect retinal neurons due to the excessive activation of the PI3K/Akt/mTOR signalling in DR. After the administration of a traditional Chinese medicine, Mingmu Xiaomeng tablets (MMXM), which have been proven as an effective mTOR inhibitor, LC3-II, p62, p-PI3K, p-Akt, and p-mTOR protein levels were significantly decreased in retinal tissue compared with that of the untreated diabetic rats. Glial fibrillary acidic protein (GFAP) is a specific glial cell marker that is used to reflect the response of glial cells under pathological conditions. The therapeutic effects of MMXM were also observed in retinal Müller cells (RMC), including the inhibition of GFAP overreaction and the restraint of local inflammation. Since RMC is a key player in inducing the expression of acute-phase response proteins and other inflammation-related genes in DR, the expressions of downstream inflammatory cytokines, such as IL-1β, IL-4, IL-6, TNF-α, and VEGF, were also significantly reduced. 

RMCs provide structural and neurotrophic support to the retina via uptake and regulation of neurotransmitters. RMC hyperplasia is one of the early pathological changes in DR [88], and upregulated GFAP expression in RMCs was found in animal models and tissue from diabetic patients. A recent study showed that mTORC1 may play an important role in RMC dysfunction during DR [89] (Table 2). Besides the upstream kinases, mTOR senses multiple stimuli, including growth factors, amino acids, nutrients, energy status, and cellular stress. Guo et al. found that high glucose (HG) treatment increased glutamine synthetase activity in the cultured RMC, which promoted the biosynthesis of Gln, leading to the activation of mTORC1 through ADP-ribosylation factor 1 (Arf 1) in Rag GTPases-dependent and Rag-independent manners [89]. RMC proliferation and activation in high-fat diet and STZ-induced mouse diabetic models were inhibited by rapamycin. Lopes de Faria et al. investigated the correlation between the autophagy machinery and ER stress in RMCs in the HG condition and found that lysosome-mediated autophagy was impaired in RMCs. However, autophagy was activated due to the sustained but insufficient ER stress response inducing the misfolded/unfolded protein under oxidative stress condition [90]. There were higher amounts of autophagosomes in the cytosol and accumulating p62/SQTSM1 cargo than those in the normal control (NC) group, which usually occurred when autophagic flux was compromised. Furthermore, the lysosome proteolytic activity decreased due to the malfunction of cathepsin L. After the administration of rapamycin, the recovery of cathepsin L activity improved the autophagic flux and reduced p62/SQTSM1 cargo accumulation leading to the amelioration of ER stress. 

Ribosomal protein S6 kinase beta-1 (S6K1) is a downstream target of the mTORC1 pathway. It is phosphorylated and activated by mTORC1; therefore; the level of p-S6 is often used as an indicator of the activation degree of the mTORC1 pathway. In the STZ-induced diabetic model, the expressions of p-S6 and VEGF were upregulated in the retina [94]. After the administration of rapamycin in HG-induced human retinal capillary endothelial cells (HRCECs), the expression of p-S6 was decreased, and the proliferation and migration of HRCECs were restrained. It indicated that mTORC1 is involved in the development of DR, targeting different cells in the retina. 

mTOR also serves as a regulator to maintain the balance between retinal neuronal death and survival based on the equilibrium between apoptosis and autophagy [97]. Due to microvascular alteration in DR, retinal neurons are subjected to ischaemia/reperfusion (I/R) injuries. Tang et al. compared autophagy level in hyperglycaemic and normoglycaemic states following I/R injury and found an elevated autophagy after two hours of ischaemia induced by middle cerebral artery occlusion (MCAO) surgery in the retinae of Akita mice [98]. After two hours of reperfusion, the expression levels of LC3B and LAMP1 were still higher in the inner retinae of Akita MCAO mice compared with sham-treated Akita mice. In contrast, the expression level of LCB3 was higher in WT sham-treated mice than WT MCAO-injured mice. It indicated that the upregulated autophagy was pre-existing in the chronic hyperglycaemic condition and sustained at a higher level in the retinal I/R episode. Moreover, the autophagy upregulation exhausted and returned to basal levels after longer time reperfusion of 22 h. It is possible that upregulated autophagy has a beneficial effect in the I/R-injured retina under hyperglycaemia. Amato et al. also found high glucose treatment significantly increased apoptosis and decreased the autophagic flux by the up-regulation of mTOR in ex vivo mouse retinal explants [92]. Compared with untreated explants, LC3 immunolabeling was dramatically reduced in different retinal layers, including GCL, INL, and OPL. After the administration of octreotide, a well-known inhibitor of the PI3K/AKT/mTOR pathway, in HG-treated retinal explants, apoptosis was reduced below control levels, and LC3 expression was increased in different types of retinal neurons, especially the bipolar cells and ganglion cells. It suggested that mTOR may play a significant role in the crosstalk between apoptosis and autophagy in DR. 

More importantly, rapamycin may have an antioxidative effect and plays a role in the amelioration of diabetic oxidative stress. Özdemir et al. found that oral rapamycin treatment reduced nitrotyrosine and malondialdehyde (MDA) levels, both of which are oxidative stress markers, in STZ-induced diabetic rat retina [99]. It is possible that rapamycin prohibits the induction of inducible NO synthase (iNOS) in the retina by reducing the expression of inflammatory mediators, such as VEGF, TNF-α, and IL-1β [100]. The inhibition for the secretions of inflammatory mediators through the AKT/mTOR pathway was further confirmed by Ran et al., who found that curcumin had comparable effects with rapamycin to inhibit the phosphorylation of AKT and mTOR, as well as reducing the HG-induced ROS in RPEC [28]. Semaglutide and rosiglitazone are two commonly used antidiabetic drugs. Yang et al. found the combined treatment of these two drugs inhibited the PI3K/Akt/MTOR signalling pathway and the inhibition of mTOR reduced oxidative stress in STZ-induced diabetic rat retina [101]. The combination administration also downregulated GFAP expression in Müller cells; however, the relationship between mTOR signalling inhibition and the alleviation of Müller cells activation is unknown.

### 4.2. mTOR and Age-Related Macular Degeneration (AMD) 

AMD is the leading cause of central vision impairment in the industrialised world [102]. There are two basic types of AMD, wet (exudative) AMD and dry (nonexudative) AMD. Wet AMD is a serious type of AMD featured with neovascularisation and subretinal haemorrhage. Most AMD patients have dry AMD, which accounts for 90% of AMD cases [103]. Dry AMD is characterised by RPE dysfunction, drusen formation, and progressive loss of neurons [104]. Although the precise mechanism of AMD has not been delineated, many studies have shown that oxidative stress acts as an initial trigger for the pathogenesis of AMD [105,106,107,108] and plays a central role in the progression of AMD [109,110,111]. As an early sign of AMD, drusen-like deposits have been found in SOD (superoxide dismutase) knockout mice [112,113]. The decreased autophagy in RPE cells exposed to oxidative stress reduces the removal of aggregated proteins and damaged organelles, leading to the formation and accumulation of those subretinal deposits [114,115]. Phospholipid decosahexaenoic acid (DHA) from the shredded photoreceptor outer segments is one of the main sources of ROS after lipid peroxidation in RPE cells [116]. The end-products of lipid peroxidation activate the nuclear factor kappa-light-chain-enhancer of activated B cells’ (NF-κB) signalling pathway, triggering a proinflammatory cascade, which could lead to choroidal neovascularization (CNV) formation [110]. 

mTOR, the key member of the PI3K/AKT/mTOR signalling pathway, plays a fundamental role in cellular nutrient, oxygen, and energy sensing [117]. A previous study has shown the strong association between hypoxia and RPE-associated neovascularisation in dry AMD [118]. The long-lasting hypoxia activates PI3K/mTOR, which increases the expression of hypoxia-inducible factor-1a (HIF-1a). The accumulation of HIF-1a protein significantly induces apoptosis and the secretion of angiogenic factors. Lin et al. found that Silibinin, a traditional medicine extract, inhibited the PI3K/mTOR signalling pathway, leading to the reduction in HIF-1a subunit accumulation, suppressing RPE apoptosis and secretion of VEGF in a rat model of VEGF-induced AMD [119]. Interestingly, Silibinin reversed hypoxia-initiated autophagy induction in hypoxia-conditioned ARPE-19 cells, although the mTOR pathway had been inhibited. This is probably because of the interaction between autophagy and oxidative stress since oxidative stress activates autophagy and elevated autophagy, in turn, reduces oxidative stress. Mitter et al. found that there was a dynamic alteration in autophagic flux in cultured RPE cells based on the time exposed to oxidative stress [115]. The autophagy activity increased significantly when exposed to short-term (4 hrs to 24 hrs) oxidative stress and decreased when exposed to long-term (1 d to 14 d) oxidative stress. Rapamycin not only protected ARPE-19 cells from an acute lethal dose of H_2_O_2_ but also rescued the autophagy activity, leading to a reduction in ROS generation and lipofuscin-like granule accumulation upon long-term oxidative stress. Besides the in vitro model, rapamycin also played a key role in attenuating an inflammatory response and oxidative stress in sodium-iodate (NaIO_3_)-induced retinal degeneration in mice as well [21].

mTOR is an essential upstream regulator of autophagy, which inhibits the ULK1-ATG13-RB1CC1/FIP200 complex. To investigate the relationship between autophagy and AMD, Yao et al. used the Cre-loxP system to knock out the Rb1cc1 gene in mice [114]. After the deletion of Rb1cc1, significant autophagy defects were observed in the RPE, including decreased conversion of LC3-I to LC3-II, accumulation of autophagy-targeted precursors, and increased numbers of mitochondria, accompanied by the deposition of inflammatory and oxidatively damaged proteins and subretinal drusenoid deposits. In contrast, Cai et al. enhanced autophagy by overexpressing miR-29, a key precursor molecule that post-transcriptionally repressed LAMPTOR1/p18 and reduced the recruitment of mTORC1 to lysosomal membranes [120]. Upon inhibition of mTORC1 activity, the elevated autophagy enhanced the removal of protein aggregates. Similar results were reported using rAAV-mTOR shRNA to block the activity of both mTOR complex 1 and 2 in the mouse laser-induced CNV model [121]. Besides the removal of protein aggregates, Ebeling et al. found that rapamycin improved the clearance of damaged mitochondria in donated human RPE cells with AMD [122]. It also showed that rapamycin increased basal respiration and attenuated mitochondrial function in RPE cells [122]. Table 3 is a summary of the in vitro and in vivo studies investigating the involvement of mTOR in AMD (Table 3).

### 4.3. mTOR and Retinitis Pigmentosa (RP)

In contrast to AMD, retinitis pigmentosa (RP) is a genetic disorder with early onset and characterised by diffuse progressive degeneration of predominantly rod photoreceptors with subsequent dysfunction of cone photoreceptors. Although this inherited retinal degeneration does not share the same common pathological processes with other ocular neurodegenerative diseases, such as AMD, DR, and glaucoma, that are induced by oxidative stress, hypoxia, and inflammation (Figure 1), mTOR plays a critical role in the pathogenesis of RP. In a rat model for retinitis pigmentosa, D’Cruz et al. found that the mutation of receptor tyrosine kinase gene Mertk caused an RPE phagocytosis defect, which led to the accumulation of rod outer segment debris [128]. More importantly, the mutation of this autophagy-related gene was found in RP patients as well [129]. As a master regulator of the autophagic signalling pathway, mTOR may also be involved in the regulation of Mertk. *MERTK* expression was shown to be regulated by rapamycin in a time-course-dependent manner [130]. In an *rd1* mouse model of retinitis pigmentosa, mTOR was upregulated in photoreceptors. Furthermore, the progression of retinal degeneration in *rd1* mice was alleviated after rapamycin treatment [131].

### 4.4. mTOR and Glaucoma

Glaucoma, a leading cause of irreversible blindness in the world, is one of the most common ocular neurodegenerative diseases. It is characterised by a progressive death of RGCs and structural damage to the optic nerve (ON) [132]. Elevated intraocular ocular pressure (IOP) has always been thought to be the major risk factor of this disease; however, RGC and nerve fibre loss may also occur in a person with normal IOP [133,134]. A great deal of studies have provided evidence showing the involvement of the mTOR signalling pathway in the pathogenesis of glaucoma. A recent study found that AMPK, a critical regulator of mTORC1, was highly expressed in RGCs from both mice with high IOP and patients with primary open-angle glaucoma [135]. Ocular hypertension-induced AMPK overexpression strongly inhibited mTORC1, leading to RGC dendrite retraction and synapse elimination in the early stage. The restoration of mTORC1 activity by knocking down AMPK rescued dendrites and synaptic contacts and promoted RGC survival. It indicated that activated mTORC1 is essential for RGC dendritic maintenance and regeneration, and the inhibition of mTORC1 may diminish its neuroprotective effects in hypertension-induced RGC injury. Similar results were found by Park et al. They observed a significant decrease in mTOR in rat glaucomatous retinas [48]. Furthermore, when autophagy was inhibited by 3-MA, apoptosis of RGCs was significantly decreased in glaucomatous retina. 

Not only in RGCs, mTOR-mediated autophagy was also activated in Müller cells in an ischaemic injury model induced by CoCl_2_ [136]. After the treatment of lutein, a potent anti-oxidant, autophagosome formation induced by rapamycin was suppressed [137]. Moreover, the rMC-1 cell viability and survival rate significantly increased when autophagy was inhibited by lutein [137]. Autophagy is generally considered as a neuroprotective mechanism in the early onset of stress condition. However, over-upregulated autophagy may exacerbate hypoxia-induced cell damage to retinal neurons. 

Owing to the versatile roles of the mTOR signalling pathway in multiple cellular functions, it could induce off-target effects [138]. The non-specific effects are also time-dependent; sustained daily rapamycin treatment may promote neuroprotection through activation of multiple pathways downstream or crosstalk with mTOR. Su et al. found that rapamycin promoted RGC survival in a rat chronic hypertensive glaucoma model via inhibition of neurotoxic mediators release and suppression of RGC apoptosis [15]. Moreover, the anti-apoptotic effects were induced directly by rapamycin instead of acting through the PI3K/AKT cell survival pathway. Rapamycin also played a role in inhibiting the activation of microglia in the glaucomatous retinas, preventing the release of pro-inflammatory factors [139]. Topical administration of rapamycin has also shown robust neuroprotective effects in a rat glaucoma model [18]. Strikingly, rapamycin eye drops could reduce IOP by inhibiting RhoA protein activation that regulates actin cytoskeleton in trabecular meshwork (TM) cells.

The trabecular meshwork, which controls the outflow of aqueous humour (AH), plays a critical role in the regulation of IOP. TM cells in the AH pathway are constantly subjected to oxidative stress, which increases the generation of intracellular reactive oxygen species (ROS), leading to mitochondrial dysfunction and apoptosis [140,141]. Besides, the TM cell is one type of post-dividing cell that does not have the capacity to remove excess harmful substances, such as damaged DNA and lipids and collagen deposits [142]. A study also showed that the autophagy homeostasis of TM cells was disrupted in glaucoma patients [143]. Decreased autophagy activity can be considered as an indication of progressive dysregulation of TM function. Studies on promoting autophagy activity in TM cells by blocking the mTOR signalling pathway have been conducted. Zhu et al. found that rapamycin treatment decreased α-actin and myocilin expression in the TM cells of a glucocorticoid-induced glaucoma (GIG) mouse, which was responsible for the extracellular matrix deposition in the TM cells [20]. Rapamycin also recovered the TM ultrastructural and morphological changes in a glaucomatous mouse model, including mitochondrial and collagen fibre arrangement and basement membrane integrity. As a result, the elevated IOP was alleviated after treatment. He et al. found that rapamycin dramatically cleared the damaged mitochondria and accumulated ROS in the TM-1 cells that were exposed to rotenone-induced oxidative stress [16]. Rapamycin also promoted mitochondrial function and prevented TM cell death. Moreover, Igarashi et al. found that topical rapamycin treatment ameliorated TM fibrosis and suppressed collagen deposition in rabbit eyes after trabeculectomy [22]. Table 4 is a summary of the in vitro and in vivo studies investigating the involvement of mTOR in glaucoma (Table 4). 

## 5. Clinical Trials of mTOR Inhibitors in Ocular Neurodegenerative Diseases

As mentioned above, rapamycin, the most established mTOR inhibitor, exhibited potent anti-angiogenic and neuroprotective effects in animal models of DR. Up to now, there have been three clinical trials that evaluated the safety and tolerability of rapamycin in patients with diabetic macular oedema (DMO). Krishnadev et al. conducted a phase I/II study that included five adult participants with diabetic macular oedema (DMO) [150]. The participants received subconjunctival sirolimus injection (440 μg) every 2 months for 12 months with the fellow eye as control. There were no significant drug-related adverse events and repeated subconjunctival injections were well-tolerated. Limited efficacy results were observed, including a 2-line improvement in visual acuity (VA) and 2 log unit decrease in retinal thickness in one participant and improvement in central retinal thickness in three participants; however, one participant had a 2-line worsening of VA and a 1 log unit increase in retinal thickness. Dugel et al. conducted a phase I study to evaluate the safety and tolerability of different dosages of sirolimus in DMO patients with two administration routes, single subconjunctival (SCJ), and intravitreal (IVT) injection, respectively (220, 440, 880, 1320, or 1760 μg vs. 44, 110, 176, 264, or 352 μg) [151]. Twenty-five DMO patients were assigned into each treatment group with the fellow eye as a control. During 90 days of observation, there were no significant drug-related adverse events and dose-limiting toxicities. For the SCJ group, a median increase in BCVA (5.0, 3.0, 4.0, and 4.0 letters) was observed at day 7, 14, 45, and 90, respectively. At day 45, the median decrease in retinal thickness was −23.7 μm. In comparison, the median increase in BCVA of IVT (2.0, 4.0, 4.0, and 4.0 letters) was observed at day 7, 14, 45, and 90, respectively. At day 45, the median decrease in retinal thickness was −52.0 μm. These clinical data provided support for prospective larger randomised trials of rapamycin in the treatment of DR. 

To date, there have been three clinical trials that evaluated the safety and efficacy of rapamycin in the treatment of AMD-associated GA. Wong et al. conducted a phase II trial that included 11 participants with bilateral GA [152]. The participants received subconjunctival sirolimus injection (440 μg) every 3 months for 24 months with the fellow eye as a control. Although the treatment was safe and well-tolerated, no significant beneficial effects of sirolimus were observed in the prevention of GA progression; a drug-associated VA decrease was instead found when compared with untreated eyes. In the phase I/II trial conducted by Petrou et al., ocular adverse events, including accelerated retinal thinning and abnormal perilesional changes, on fundus autofluorescence (FAF) were found in two of six participants besides drug-related endophthalmitis [153]. Later, a larger phase II trial was conducted, which included 52 participants with GA treated with monthly intravitreal injection of sirolimus (440 μg) [154]. The trial was suspended because of the observed sterile endophthalmitis in three participants treated with sirolimus. No significant structural or functional benefits were observed after sirolimus injection when compared with the sham group. 

There are several potential reasons for the unsatisfactory efficacy in the clinical trial of rapamycin for GA. Firstly, the neuroprotective effects of rapamycin that are efficient in the experimental models of AMD may not be potent enough to prevent and slow down the progression of GA alone, especially in its later stages. Secondly, the protective effect induced by upregulated autophagy in early AMD may exacerbate the apoptosis of retinal neurons in the late stage of AMD. Furthermore, due to the complexity of the mTOR signalling pathway, its effects to modulate the pathological process of disease is unpredictable, and off-target effects are inevitable, especially in the long-term treatment with rapamycin. A systemic kinome-wide approach is required to profile the selectivity and potency of mTOR inhibitors. Recently, Liu et al used chemical proteomics and assays to study the enzymatic activity, protein binding, and disruption of cellular signalling of some mTOR inhibitors, including Torin 1, PP242, Ku-0063794, and WYE354 [155]. The mTOR pathway also has a different contribution in the retinal neurodegeneration in different pathological contexts. Although chronic ischaemic changes are the common pathological pathway of glaucoma and DR, and RGCs are under energetic stress due to ischaemia, rapamycin-induced autophagy played a positive role in promoting RGCs’ survival in the diabetic retinas, whereas increased RGC apoptosis was found in the glaucomatous retinas with rapamycin. AMD is a complex disease with a distinct pathological context in different stages of the disease. The modulation of rapamycin may result in paradoxical outcomes in different types of AMD as well. Indeed, clinical trials of mTOR inhibitors in CNV have also been performed with more favourable results. 

A series of pilot clinical trials have been conducted to evaluate the safety and efficacy of mTOR inhibitors for CNV both as a single drug and co-treatment with anti-VEGF therapy. Nussenblatt et al. performed a phase I/II clinical trial that included three CNV patients receiving an oral dose of rapamycin (2 mg daily) combined with intravitreal anti–VEGF injection [156]. The treatment was safe and well-tolerated and there were no systematic drug-related adverse events during the six-month observation. Compared with other immunosuppressive drugs, including daclizumab and infliximab, there was no significant difference in the reduction in anti-VEGF injection frequency nor VA improvement and retinal structure amelioration. Furthermore, a recent phase II trial was performed in 2021 to evaluate the safety of the monotherapy with intravitreal sirolimus, and its efficacy was compared with conventional anti-VEGF treatment in exudative AMD [157]. Twenty participants with CNV were assigned to each treatment group with the fellow eye as control. No obvious adverse events were observed, and the treatment is safe and tolerable. VA improvement (6 letters) was observed for both treatment groups; however, there was no significant difference between each other. Most importantly, significant anatomic improvement was found after sirolimus treatment. The mean central subfield thickness (CST) was decreased by 40 μm in the sirolimus group compared with the 20 μm CST increase in the anti-VEGF group. The second-generation mTOR inhibitor, Palmoid 529, was also tested in a clinical trial for the treatment of CNV. Dalal et al. conducted a phase I trial that included 13 CNV patients to assess the safety and efficacy of Palmoid 529 subconjunctival injection (1.9 mg, every four weeks) for 12 weeks in a short period [158]. There were no drug-related adverse events and no ocular or systemic safety concerns for the treatment. Probably due to the limited sample size, no treatment effects were found in those anti-VEGF refractory patients. Larger-scale randomised studies are, therefore, required to test the efficacy of the dual inhibitor in the treatment of CNV. Table 5 is a summary of the clinical trials of mTOR inhibitors in ocular degenerative diseases including AMD and DR (Table 5).

## 6. Discussion

**Figure 4:**
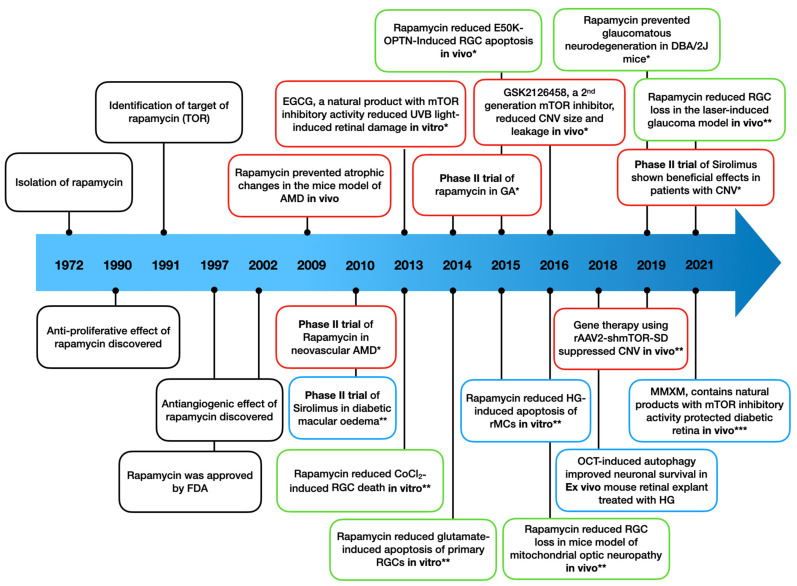
Timeline of mTOR inhibitors from discovery to the clinic for the treatment of ocular neurodegenerative diseases. Abbreviations: AMD, age-related macular degeneration; CNV, choroidal neovascularization; EGCG, epigallocatechin gallate; HG, high glucose; GA, geographic atrophy; MMXM, Mingmu Xiaomeng; OCT, octreotide; OPTN, optineurin; RGC, retinal ganglion cell; rMCs, retinal Müller cells. References in the figure: 1992 [55], 1990 [60], 1991 [1], 1997 [63], 2002 [61], 2009 [159], 2010 * [162], 2010 ** [161], 2013 * [160], 2013 ** [123], 2014 * [146], 2014 ** [16], 2015 * [44], 2015 ** [94], 2016 * [120], 2016 ** [83], 2018 [89], 2019 * [19], 2019 ** [136], 2021 * [153], 2021 ** [28], 2021 *** [91].

In line with the potent antioxidant and anti-inflammatory effects of mTOR inhibitors in different ocular neurodegenerative disease models shown above, rapamycin, a lead mTOR inhibitor, presents an attractive treatment option in the clinical trials of DR and AMD with a favourable safety profile and sustained ocular pharmacokinetics (Table 5). However, some studies reported that mTOR activation may have beneficial effects on the survival of cellular components in the retina. Cao et al showed that NGF (nerve growth factor) protected RPE cells against H_2_O_2_-induced cell apoptosis through the PI3K/Akt/mTOR and ERK/MARK signalling pathway [23]. Co-treatment with rapamycin diminished NGF-induced S6 phosphorylation and protective effects against oxidative stress in ARPE-19 cells. Since the modulation of mTOR was conducted in an RPE cell line, it is possible that mTOR upregulation may induce distinctive effects in vivo. mTOR may also have different contributions to different cellular components in the retina and to different disease conditions, respectively. Park et al. reported that the upregulation of mTOR decreased RGC apoptosis in glaucomatous retinas, which was instead increased in the diabetic retinas [48]. mTOR-mediated autophagy may, therefore, play different roles in RGCs’ survival in different disease conditions. It is unclear whether prolonged and/or overregulated autophagy may have detrimental effects to the retinal cell survival. Especially, the mTOR signalling pathway may also affect the metabolism in the retina. Fang et al. found that short-term rapamycin treatment (6 weeks) induced metabolic impairment in mice, but prolonged rapamycin treatment (20 weeks) reversed the detrimental effects, with better metabolic profiles, increased oxygen consumption and ketogenesis, and markedly enhanced insulin sensitivity [162]. Yet, conflicting results were reported; short-term hyperglycaemia (1 month) upregulated mTORC1 activity, inhibited autophagy, and prevented RGCs death, whereas prolonged hyperglycaemia (6 months) downregulated mTORC1 activity, promoted autophagy, and induced RGCs damage [93]. In light of this, it is important to address how mTOR signalling contributes to retinal neurodegeneration. A systematic profiling of mTOR signalling has been conducted in the foetal fibroblasts [163], but there is no publication covering the mTOR genetic profile in the disease model of retinal neurodegeneration. With more evidence on how mTOR modulates autophagy, cell proliferation, apoptosis, and metabolism in the retina, precise treatment using new drug delivery techniques and gene therapy may avoid adverse effects and provide higher therapeutic effectiveness. Indeed, rAAV-mTOR shRNA (recombinant adeno-associated virus-delivered mTOR inhibiting short hairpin RNA) treatment significantly reduced CNV lesions and decreased local inflammation in a laser-induced mouse model [121]. Another gene therapy study using rAAV2-shmTOR-SD achieved similar results [127]. Furthermore, the development of newer compounds that selectively induce or target autophagy may have a more promising therapeutic perspective in ocular neurodegenerative diseases (Figure 4). Wen et al. found that inhibition of mTORC2 alone resulted in blood–optic nerve barrier disruption, but co-treatment with rapamycin and mTORC2 activator SC79 improved RGC survival [164].

## 7. Conclusions

Autophagy is an essential catabolic process critical for stress responses and the maintenance of cellular homeostasis. Autophagy promotes cell survival by eliminating damaged cellular components in response to oxidative stresses. As one of the key regulators of autophagy, the involvement of the mTOR signalling pathway in the pathophysiology of major ocular neurogenerative diseases, including DR (Table 2), AMD (Table 3), glaucoma (Table 4), and RP, was summarised in this review, which focused on the common pathological processes, including mitochondrial dysfunction, elevated ROS level, and increased ER stress induced by oxidative stress, hypoxia, and inflammation (Figure 1). Each of these processes plays a substantial role in the regulation of mTOR by modulating the upstream signalling pathways, such as PI3K/Akt, AMPK, and TSC1/2. Although rapamycin may be an attractive treatment option in DR and AMD, more clinical trials are still needed. It is also essential to understand how mTOR modulates autophagy, cell proliferation, apoptosis, and metabolism in the retina. New drug delivery techniques and gene therapy, as well as selective regulators in the mTOR pathway, may help to avoid the adverse effects and provide more precise treatment, yielding higher therapeutic efficacy. 

## Figures and Tables

**Table 1:** The potency, specificity, and adverse effects of mTOR inhibitors.

Drug	Target	Potency (IC_50,_ nM)	Pros/Cons	Development Status	Adverse Effects
1st generation mTOR inhibitors					
Rapamycin	mTOR/FKBP12	0.1	1st FDA-approved mTOR inhibitor/ low biological utilisation due to its poor water solubility and stability	FDA-approved	Hyperglycaemia, fatigue, nausea/vomiting, anaemia, stomatitis, mucositis, pulmonary and metabolic toxicities [54,55,56]
Temsirolimus	mTOR/FKBP12	1.76	Relatively high water solubility and stability, intravenous administration only	FDA-approved	
Everolimus	mTOR/FKBP12	1.6–2.4	Relatively high water solubility and stability, low toxicity and high efficacy for some types of tumours	FDA-approved	
Ridaforolimus	mTOR/FKBP12	0.2–5.6	Latest developed rapalogs, well-tolerated in children	FDA-approved	
2nd generation mTOR inhibitors					
Torin1	mTORC1/mTORC2	0.29 (mTORC1)/5 (mTOR)	Strong anti-proliferation activity/poor stability and low oral bioavailability	Preclinical	Hyperglycaemia, fatigue, nausea/vomiting, stomatitis, mucositis, diarrhoea, decreased appetite, liver dysfunction, pneumonia [57,58,59]
PP242	mTORC1/mTORC2	8 (mTOR)	Relatively strong selectivity to mTOR	Preclinical	
AZD8055	mTORC1/mTORC2	10 (mTORC1)/2.8 (mTOR)	Potent anti-proliferation and apoptosis induction activity/relatively high liver toxicity	Phase I	
OSI-027	mTORC1/mTORC2	4 (mTORC1)/22.6 (mTOR)	Strong inhibitory effects on mTOR, dose-dependent manner in patients with some types of tumours	Phase I	
PI-103	mTOR/PI3K	3–3.6 (PI3K)	1st developed mTOR/PI3K dual inhibitor/poor drug properties	Preclinical	Hyperglycaemia, fatigue, nausea/vomiting, mucositis, diarrhoea, decreased appetite, rash [60,61,62]
GSK2126458	mTOR/PI3K	0.18 (mTORC1)/0.019–0.13 (PI3K)	Confirmed target engagement in blood and lungs/affect insulin release and blood glucose level	Preclinical	
NVP-BEZ235	mTOR/PI3K	mTOR (20.7)/4–75 (PI3K)	Potent PI3K inhibitory effects on PI3K	Phase I	

**Table 2:** Involvement of mTOR in DR: in vitro and animal studies.

Target Cells or Tissue	Disease Model	mTOR Regulator	Autophagy-Related Markers	Related Pathways	Effects of Regulated mTOR	References
R28 cells	Hypoxia-induced AMD model	Insulin	LC3A↓	PI3K/AKT/mTOR↑	Oxidative stress↓ VEGF↓	[49]
rMC-1	HG	Rapamycin	Beclin1↑ p62↓	mTOR↓	Apoptosis↓ VEGF↓	[90]
ARPE-19	HG	Curcumin	-	PI3K/AKT/mTOR↓	TNF-α/ IL-1β/IL-6↓	
661W cells	HG	3-MA	LC3B2↓ p62↑	PI3K/AKT/mTOR↑	ROS↑ Mitophagy↓ Apoptosis↑	[91]
Ex vivo mouse retinal explants	HG	Octreotide	LC3-II↑ LC3-II net flux↑	mTOR/S6K1↓	Apoptosis↑	[92]
RGCs	STZ-induced diabetic rats	3-MA	LC3B↓ Beclin-1↑	AMPK↓/mTOR↑	Apoptosis↑	[28]
RGCs	STZ-induced diabetic mice	Rapamycin	-	mTOR/S6K1↓	GLUT1↓ GFAP↓	[93]
RMCs	STZ-induced diabetic rats/HG	PPP1CA	-	YAP/GS/Gln/ mTORC1↑	RMCs activation/ proliferation↑	[89]
RMCs	STZ-induced diabetic rats	MMXM	LC3-II↑ p62↓	PI3K/AKT/mTOR↓	IL-1β/ IL-6↓ VEGF↓ GFAP↓	[87]
Retina tissue	STZ-induced diabetic rats	Rapamycin	-	mTORC1/S6K1↓	VEGF↓ PEDF↓ HRCECs proliferation/migration↓	[94]
Retina tissue	STZ-induced diabetic rats	Phosphatidic acid	-	mTOR/S6K1↑	Apoptosis↓	[95]
Retina tissue	STZ-induced diabetic rats/Ins2^Akita^ mice	Insulin/phloridzin	-	AKT/mTORC2↑ mTORC1/S6K1/ 4E-BP1 ↔	Retinal protein synthesis↑	[96]

**Table 3:** Involvement of mTOR in AMD: in vitro and animal studies.

Target Cells or Tissue	Disease Model	mTOR Regulator	Autophagy-related Markers	Related Pathways	Effects of Regulated mTOR	Reference
ARPE-19	H_2_O_2_-induced RPE cell injury model	Silibinin	LC3A↓	PI3K/AKT/mTOR↓	Oxidative stress↓ VEGF↓	[46]
ARPE-19/hRPE	H_2_O_2_-induced RPE cell injury model	a-MSH	-	PI3K/AKT/mTOR↑	Oxidative stress↓ Apoptosis↓	[123]
hRPE/HUVEC	Hypoxia-induced RPE cell injury model	Temsirolimus	-	mTOR↓	VEGF↓ PEDF↓	[19]
hRPE	Human AMD patient	Rapamycin	LC3-II/I↑	mTOR↓	Mt function↑ Mitophagy↑	[122]
ARPE-19/hRPE	H_2_O_2_-induced RPE cell injury model (acute/chronic)	Rapamycin	LC3 puncta↑	mTOR↓	Oxidative stress↓ ROS↓ Lipofuscin-like deposit↓	[115]
ARPE-19	H_2_O_2_-induced RPE cell injury model	Resveratrol	LC3-II/I↑ p62↓	mTOR↓	Apoptosis↓ VEGFA↓ IL-6/ IL-8↓	[124]
ARPE-19	Lipid-peroxidation-induced RPE injury model	Glucosamine	LC3-II/I↑ p62 ↗↘	AMPK↑/mTOR↓	Lipofuscin-like deposit↓	[125]
ARPE-19/hRPE	αB-crystallin R120G-mutation-induced protein aggregation model	miR-29	LC3-II/I↑ p62↓	mTOR↓	Protein aggregation↓	[120]
Retina tissue	Laser-induced model of CNV	GSK2126458	-	PI3K/mTOR↓	Vascular leakage↓ CNV lesions↓ Apoptosis↓ Serum glucose level↑	[126]
Retina tissue	Laser-induced model of CNV	rAAV-mTOR shRNA	LC3B↑ ATG7↑	PI3K/mTOR↓	Vascular leakage↓ CNV lesions↓ Apoptosis↓	[127]
Retina tissue	Laser-induced model of CNV	rAAV2-shmTOR-SD	-	mTOR↓	CNV lesions↓ Apoptosis↓	[127]
Retina tissue	NaIO_3-_induced retinal degeneration	Rapamycin	-	mTOR↓	Oxidative stress↓ Apoptosis↓ GFAP↓ IL-6/ MCP-1/TNF-α↓	[21]

**Table 4:** Involvement of mTOR in glaucoma: in vitro and animal studies.

Target Cells or Tissue	Disease Model	mTOR Regulator	Autophagy-Related Markers	Related Pathways	Effects of Regulated mTOR	References
NSC-34 /661W cells	2bpIns-OPTN-induced cell death	Rapamycin	LC LC3-II/I↑↑ LC3↑ ATG5↑	mTOR↓	Apoptosis↓ ER stress↓	[144]
TM-1 cells	Rotenone-induced oxidative stress model	Rapamycin	LC3-II/I↑ p62↓	PI3K/AKT/mTOR↑	Apoptosis↓ Oxidative stress↓Mitophagy↑	[16]
RGC-5	E50K-OPTN-induced RGC death	Rapamycin	-	mTOR↓	Apoptosis↓	[145]
Retina tissue/RGC-5	Rat CoCl2-induced hypoxia model	Rapamycin	-	mTOR/RhoA/ROCK↓	IOP↓ RGCs loss↓ Microglial activation↓Mitophagy↑	[119]
HCF cells/TM cells	TGFβ1-induced fibrosis/rabbit model of glaucoma filtration surgery	Rapamycin/Torin-1	-	AKT/mTOR↓	HCF proliferation/migration↓ TM fibrosis↓	[22]
RGCs/TM cells	Mouse glucocorticoid-induced glaucoma model	Rapamycin	LC3-II/I↑ Beclin-1↑ p62↓	mTOR↓	IOP↓ RGCs loss↓ TM fibrosis↓ Mitophagy↑	[20]
RGCs	Mouse chronic hypertensive glaucoma model	Rac1 cKO	LC3-II/I↑ Beclin-1↑ p62↓	mTOR↓	Apoptosis↓ RGCs loss↓	[146]
RGCs	Rat hypertensive glaucoma model	3-MA	LC3B↓ Beclin-1↓	AMPK↓/mTOR↑	Apoptosis↑	[48]
RGCs	Rat hypertensive glaucoma model	Rapamycin	LC3-II↑ p62↓	mTOR↓	Axon loss↓	[147]
RGCs	E50K-OPTN-induced normal tension glaucoma model	Rapamycin	LC3↑ p62↓	mTOR↓	Apoptosis↓ Axon loss↓	[148]
RGCs	DBA 2J mouse model for experimental glaucoma	Rapamycin	-	mTOR↓	Apoptosis↓ Axon loss↓	[26]
RGCs	Rat microbead occlusion model/ex vivo rat glaucoma model	Rapamycin	LC3-II/I↑ p62↓	mTOR↓	Apoptosis↓ RGCs loss↓	[18]
RGCs	E50K-OPTN-induced RGC death	Rapamycin	LC3-II↑ p62↓	mTOR↓	Apoptosis↓ RGCs loss↓ TDP-43 aggregation↓	[43]
RGCs	Rat laser-induced glaucoma model	Rapamycin	-	mTORC1/S6K1↓	Apoptosis↓ RGCs loss↓ VEGFR-2↓	[24]
RGCs	Mouse microbead occlusion model	Rapamycin	-	AMPK↑/mTOR↓	RGCs loss↓	[135]
RGCs	Circumlimbal-suture-induced OHT rat model	Rapamycin	LC3-II/I↑ p62↓	AMPK↑/mTOR↓	Apoptosis↓ RGCs loss↓	[149]
BV2 microglia/primary RGCs/retina tissue	Rat chronic hypertensive glaucoma model	Rapamycin	-	AKT↔/mTOR↓	Apoptosis↓ iNOS/TNF-a/NF-kB↓ Microglial activation↓	[15]
Retina tissue	Ndufs4 KO mouse model of mitochondrial optic neuropathy	Rapamycin	-	mTOR↓	Apoptosis↓ Microglial activation↓ Inflammation↓	[88]

**Table 5:** Summary of clinical trials of mTOR inhibitors in ocular neurodegenerative diseases.

Study (NCT Number)	Design	Subjects	Intervention	Treatment Regimen	Results	Reference
Phase II trial Naor et al. 2010 (NCT00656643)	Four-arm study in US; placebo injection as control	131 with diabetic macular oedema	Sirolimus subconjunctival injection	Two subconjunctival injections of 220, 440, 880 μg, or placebo (1:1:1:1) observation through day 180	Awaiting results	[159]
Phase I/II trial Krishnadev et al. 2011 (NCT00711490)	Single-arm study in US; fellow eye as control	5 with diabetic macular oedema	Sirolimus subconjunctival injection	440 μg injection every 2 months for 12 months follow-up period	Safe and well-tolerated; efficacy trials required	[150]
Phase I trial Dugel et al. 2012 (NCT00401115)	Two-arm study in US; fellow eye as control	50 with diabetic macular oedema (*n* = 25 for SCJ and IVT, respectively)	Sirolimus single subconjunctival (SCJ)/intravitreal injection (IVT)	SCJ (220, 440, 880, 1320, or 1760 μg)/IVT (44, 110, 176, 264, or 352 μg); observation through day 90	Safe and well-tolerated (no dose-limiting toxicities); efficacy trials required	[151]
Phase I/II trial Naor et al. 2010 (NCT00712491)	Two-arm study in US; fellow eye as control	20 with AMD (CNV); *n* = 10 for each arm	Rapamycin intravitreal injection	Three injections of 352 or 1320 μg observation through 12 months	Awaiting results	[160]
Phase II trial Nussenblatt et al. 2010 (NCT00304954)	Four-arm study in US; fellow eye as control	13 with AMD (CNV)	Intravenous daclizumab/intravenous infliximab/oral rapamycin/observation with anti-VEGF therapy	Daily 2 mg oral tablet (*n* = 3) vs. daclizumab, vs. infliximab vs. no immunosuppression plus intraocular anti-VEGF therapy for 6 months fellow up	Safe and well-tolerated; no benefit	[156]
Phase II trial Abraham et al. 2010 (NCT00766337)	Three-arm study in US; placebo comparator as control	62 with AMD (CNV)	Sirolimus in combination with ranibizumab subconjunctival injection	440 or 1320 μg both with 500 μg ranibizumab every 2 months for 24 months fellow up	Awaiting results	[161]
Phase II trial Wong et al. 2013 (NCT00766649)	Single-arm study in US; fellow eye as control	11 with AMD (GA)	Rapamycin subconjunctival injection	440 μg injection every three months for 24 months follow-up	Safe and well-tolerated; no benefit	[152]
Phase I trial Dalal et al. 2013 (NCT01271270)	Single-arm study in US; fellow eye as control	13 with AMD (CNV)	Palomid 529 subconjunctival injection	1.9 mg injection every 4 weeks for 12 weeks follow-up	Safe and well-tolerated; efficacy trials required	[158]
Phase I/II trial Petrou et al. 2014 (NCT01445548)	Single-arm study in US; fellow eye as control	6 with AMD (GA)	Rapamycin intravitreal injection	440 μg injection every two months for 12 months follow-up	Ocular adverse events appeared; no benefit	[153]
Phase II trial Gensler et al. 2017 (NCT01675947)	Two-arm study in US; sham treatment as control	52 with AMD (GA); *n* = 27 for rapamycin	Rapamycin intravitreal injection	440 μg injection monthly for 24 months follow-up	Safe and well-tolerated; no benefit	[154]
Phase II trial Minturn et al. 2021 (NCT02357342)	Two-arm study in US; fellow eye as control	40 with AMD (CNV); *n* = 20 for each arm	Sirolimus intravitreal injection/anti-VEGF therapy	440 μg injection every two months for 6 months follow-up	Safe and well-tolerated; CST decreased by 40 μm in sirolimus group (*p* = 0.03)	[157]
